# *Mycobacterium tuberculosis* Cpn60.2 (GroEL2) blocks macrophage apoptosis via interaction with mitochondrial mortalin

**DOI:** 10.1242/bio.023119

**Published:** 2017-03-13

**Authors:** Sunil Joseph, Alex Yuen, Vijender Singh, Zakaria Hmama

**Affiliations:** Division of Infectious Diseases, Department of Medicine, Vancouver Costal Health Research Institute, University of British Columbia, Vancouver, British Columbia V6H 3Z6, Canada

**Keywords:** Host-pathogen interaction, Phagosome, Intra-cellular trafficking, Mycobacterial persistence

## Abstract

Earlier studies suggested that *Mycobacterium tuberculosis* (Mtb) proteins exported within the host macrophage play an essential role in tuberculosis pathogenesis. In fact, Mtb proteins interact with and deactivate key regulators of many macrophage functions such as phago-lysosome fusion and antigen presentation, resulting in the intracellular persistence of pathogenic mycobacteria. Cpn60.2 is an abundant Mtb chaperone protein, restricted to cell cytoplasm and surface, that was reported to be essential for bacterial growth. Here, we provide evidence that once Mtb is ingested by the macrophage, Cpn60.2 is able to detach from the bacterial surface and crosses the phagosomal membrane towards mitochondria organelles. Once there, Cpn60.2 interacts with host mortalin, a member of the HSP 70 gene family that contributes to apoptosis modulation. In this regard, we showed that Cpn60.2 blocks macrophage apoptosis, a phenotype that is reversed when cells are pretreated with a specific mortalin inhibitor. Our findings have extended the current knowledge of the Mtb Cpn60.2 functions to add a strong anti-apoptotic activity dependent on its interaction with mitochondrial mortalin, which otherwise promotes Mtb survival in the hostile macrophage environment.

## Abstract (precis)

**Summary:** Once macrophage ingests *Mycobacterium tuberculosis*, the chaperone Cpn60.2 detaches from mycobacterial surface and traffics towards mitochondria. Once there, Cpn60.2 interacts with host mortalin leading to inhibition of macrophage's apoptosis.

## INTRODUCTION

A key feature of tuberculosis (TB) pathogenesis is the persistence and replication of *Mycobacterium tuberculosis* (Mtb) bacilli in alveolar macrophages, which usually act as an efficient first line of defense against inhaled pathogens (Eddens and Kolls, 2012). While many respiratory pathogens, such as *Streptococcus* and *Mycoplasma*, express their pathogenic effects by means of a single powerful toxin (Barnett et al., 2015; Tully, 1981), substantial research efforts demonstrated that instead Mtb secretes, within the host macrophage, a variety of proteins and glycolipids which act in concert to deactivate essential macrophage functions. In this context, earlier studies showed that mycobacterial 19 kDa lipoprotein causes transcriptional downregulation of MHC class II molecules to prevent antigen presentation to T helper cells (Noss et al., 2001). Thereafter, the surface glycolipid lipoarabinomannan (LAM) was shown to block cytosolic Ca^2+^-dependent activation of phosphatidylinositol 3 kinase hVPS34, essential for the production of phosphatidylinositol 3 phosphate (PI3P) on phagosomes. PI3P facilitates membrane docking for the effector protein early endosome antigen 1 (EEA-1) (Vergne et al., 2003). LAM action can be further amplified by secreted acid phosphatase (SapM), which depletes phagosomal PI3P (Vergne et al., 2005). More recent studies revealed that protein tyrosine phosphatase A (PtpA) targets the subunit H of vacuolar-H^+^-ATPase (V-ATPase) complex on phagosomal membrane to prevent luminal acidification (Wong et al., 2011). Nucleoside diphosphate kinase (Ndk) is another important Mtb virulence factor that expresses GTPase-Activating Protein (GAP) activities towards phagosomal Rab5 and Rab7 (Sun et al., 2010), and by doing so prevents Rab5 and Rab7 interaction with EEA-1 and Rab-interacting lysosomal protein (RILP), respectively, and aborts phagosome maturation (Sun et al., 2010). While these findings, and others (reviewed in Cambier et al., 2014), represent a tremendous progress toward the knowledge of molecular and subcellular mechanisms of Mtb interaction with the host cell, many other mycobacterial proteins remain to be investigated in order to establish a full picture of TB pathogenesis. In this regard, one of the most abundant, albeit less characterized, Mtb proteins is the chaperone Cpn60.2 (GroEL2, Hsp65) (Kong et al., 1993). Like the 19 kDa lipoprotein, Cpn60.2 and its closely related Cpn60.1 chaperone localize within the outer layer of Mtb cell wall (Stokes, 2013). Cpn60.1 was found to be dispensable but deletion of Cpn60.2 is lethal (Hu et al., 2008), suggesting a key role in the biogenesis of critical Mtb proteins. Beside its contribution to bacterial uptake via interaction with surface molecule CD43 (Hickey et al., 2010), very little is known about the biological properties of Cpn60.2 towards macrophages.

Even though both Cpn60s are surface molecules, only Cpn60.1 is generally detectable in standard Mtb culture media (Cehovin et al., 2010). However, a recent study revealed an association between Mtb virulence and secretion of Cpn60.2 (Vargas-Romero et al., 2016), consistent with earlier studies revealing the presence of Cpn60.2 in the cerebrospinal fluid of TB meningitis patients (Mudaliar et al., 2006). Taken together, these observations suggest that Cpn60.2 might contribute to Mtb evasion of macrophage innate immunity. The present study verified this hypothesis and demonstrated that Mtb is able to export Cpn60.2 beyond the phagosomal membrane towards mitochondria organelles to interfere with mitochondrion-regulated apoptosis. We also demonstrated that apoptosis inhibition by Cpn60.2 is dependent, at least in part, on its interaction with the mitochondrial chaperone, mortalin.

## RESULTS AND DISCUSSION

### *M. tuberculosis* exports Cpn60.2 into macrophage cytosol

**Fig. 1.**
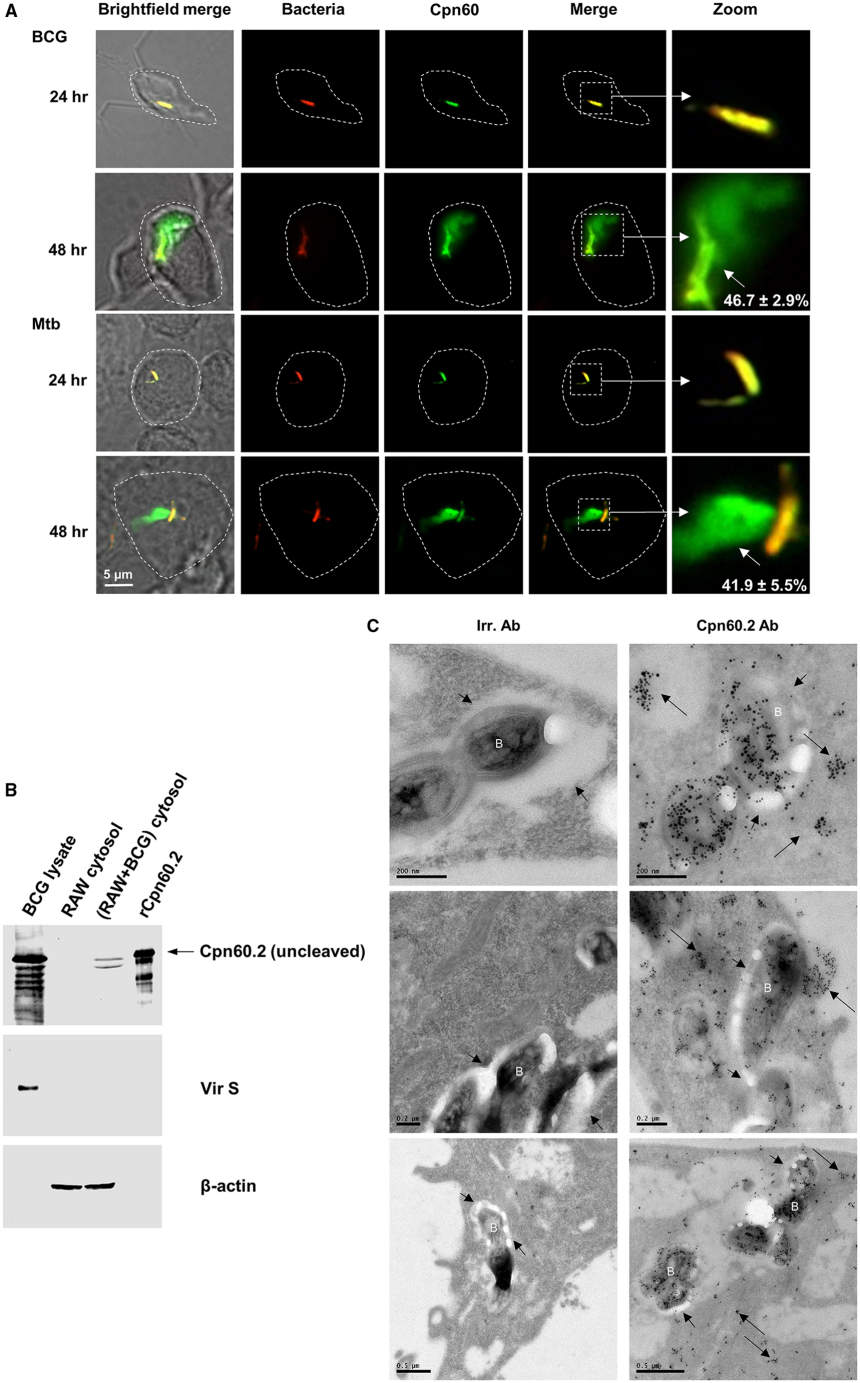
**Cpn60.2 exits phagosomal membrane in BCG- and Mtb-infected macrophages**. (A) RAW macrophages were infected with red-fluorescent-BCG and -Mtb (MOI, 20:1) for the indicated time periods. Cells were then stained with Cpn60.2 antibody (1:100) and FITC-conjugated goat anti-rabbit IgG (1:3000) (green fluorescence) and analyzed by confocal microscopy. Yellow signal in merged images (4×magnification panels) indicates bacteria-associated Cpn60.2 while green signal (short arrows) indicates Cpn60.2 diffusion beyond phagosomes. Dotted lines indicate the macrophage cell boundary. Values are means±s.d. of diffused Cpn60.2 observed in 50-60 cells from three independent experiments. (B) Cytosolic fractions from uninfected or BCG-infected macrophages were subjected to SDS-PAGE along with BCG lysate (2 µg) and rCpn60.2 (60 ng) and western blotted with Cpn60.2 antibody (1:500). Membranes were revealed with AF680-conjugated goat anti-rabbit IgG (1:10,000). Blots were then stripped, re-probed with Vir S antibody (1:1000) to control for the bacterial contamination (middle panel) and and β-actin antibody (1:1000) to control for equal protein loading (lower panel). (C) Mtb-infected macrophages were subjected to immunogold staining with control irrelevant antibody (Irr. Ab, left image) or Cpn60.2 antibody at 1:50 (right image) and revealed with ultra-small goat anti-rabbit IgG (1:50) as described (Sun et al., 2013). Long arrows indicate translocated Cpn60.2 into macrophage cytosol whereas short arrows denote the phagosomal membranes surrounding the phagosome-enclosed bacteria (marked as ‘B’). Data in A and B are representative of three independent experiments.

A recent study demonstrated that mycobacterial serine protease Hip1 converts cell wall-associated Cpn60.2 into secreted monomeric subunits in response to stress conditions within the macrophage, and the cleavage occurs between Arg12 and Gly13 residues at the N-terminus of Cpn60.2 (Naffin-Olivos et al., 2014). These findings suggest that Cpn60.2 subunits in the phagosome might translocate to the cytosol and disturb essential macrophage functions. To verify this hypothesis, we first performed confocal microscopy analyses of Mtb- and BCG-infected macrophages stained for intracellular Cpn60.2. Images obtained showed that at 24 h post-infection, Cpn60.2 staining remains limited to intra-cellular bacteria (Fig. 1A). However, at the 48 h time-point, an abundant green fluorescence signal was observed at a far distance from ingested BCG organisms (46.7±2.9%) and Mtb (41.9±5.5%), suggestive of possible secretion and export of Cpn60.2 beyond phagosomes (Fig. 1A). Staining of uninfected cells showed that the anti-Cpn60.2 antibody is not cross-reacting with host Hsp60 (data not shown). We have selected the 48 h time point for further experiments and prepared soluble lysate fractions from BCG-infected macrophages for western blot analyses, which revealed the presence of Cpn60.2 in macrophage cytosol (Fig. 1B, upper panel). To rule out the possibility that BCG gets broken during macrophage lysate preparation leading to a leakage of Cpn60.2, blots were subsequently reprobed with antibody to Vir S, which is a non secreted mycobacterial protein (Mawuenyega et al., 2005). Results in Fig. 1B (middle panel) shows that Vir S is undetectable in the cytosolic fraction of BCG-infected cells. Since Cpn60.2 is a known Hip1 substrate, the protease activity of Hip1 leads to the cleavage of Cpn60.2 in the infected macrophages (Naffin-Olivos et al., 2014; Rengarajan et al., 2008). Multiple Cpn60.2 bands in the western blot represent uncleaved and cleaved forms, respectively. Recombinant Cpn60.2 protein is also reported to show autoproteolysis (Qamra and Mande, 2004) causing multiple banding pattern in the immunoblot. Thereafter, deeper EM investigations of Mtb infected macrophages provided clear-cut evidence for massive Cpn60.2 translocation from the phagosome into the cytosolic compartment (Fig. 1C). Taken together, these data demonstrate that mycobacteria are able to export the chaperone Cpn60.2 (molecular weight, 65 kDa) beyond their phagosomal membrane, consistent with earlier evidence that mycobacterial proteins up to 70 kDa are able to exit phagosomes (Teitelbaum et al., 1999).

### Cpn60.2 translocates to macrophage mitochondria

**Fig. 2.**
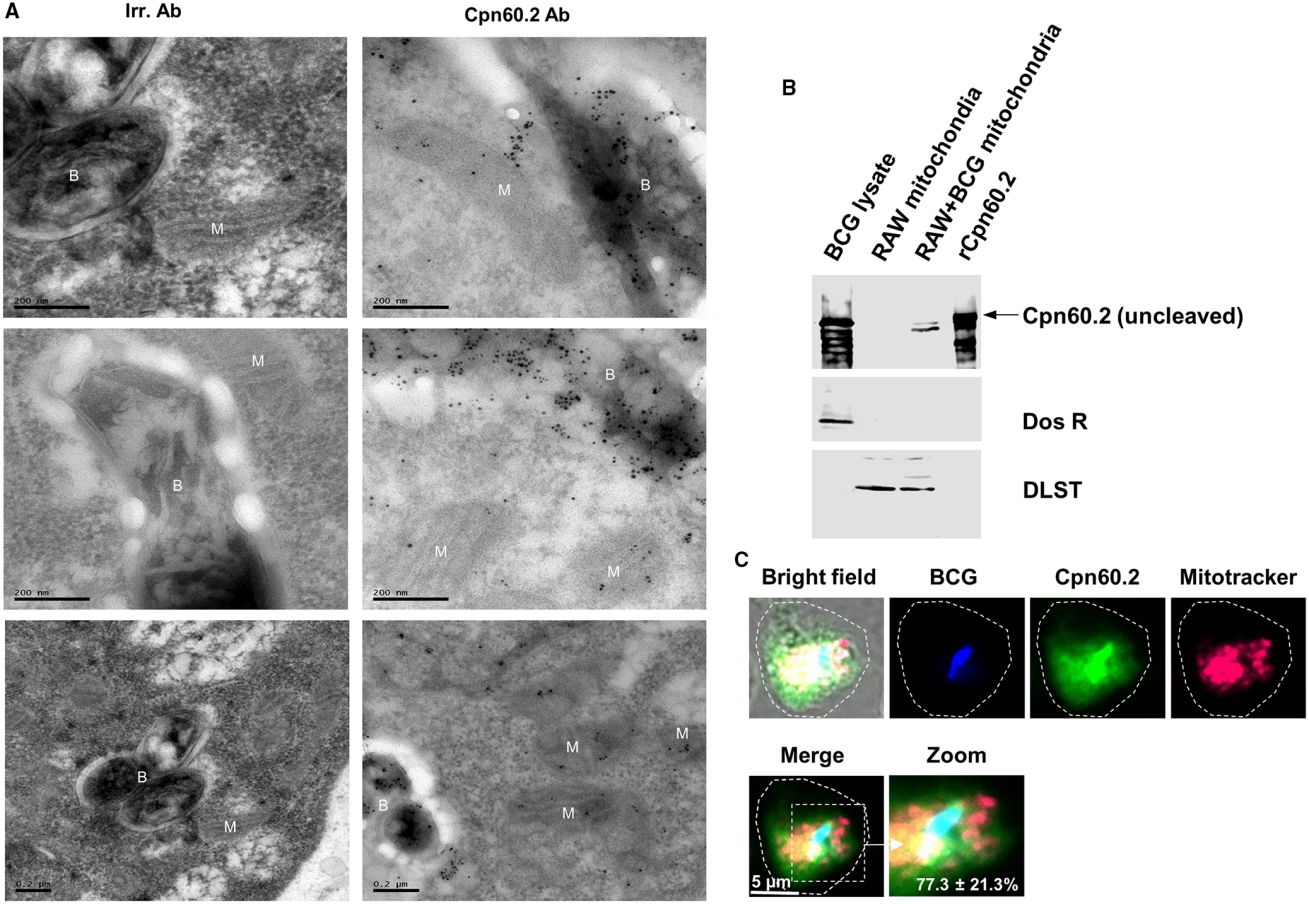
**Cpn60.2 reaches macrophage mitochondria.** (A) EM grids from Fig. 1C were re-examined to localize mitochondria. The intracellular mycobacteria and macrophage mitochondria are denoted ‘B’ and ‘M’, respectively. Black dots inside mitochondria indicate the translocated Cpn60.2 inside mitochondria of infected cells. (B) Mitochondrial fractions from uninfected and BCG-infected macrophages along with BCG lysate (2 µg) and rCpn60.2 (60 ng) were subjected to western blotting with Cpn60.2 antibody as in Fig. 1B. Membranes were stripped and re-probed with Dos R antibody (1:1000) and mitochondrial Dihydrolipoamide S-succinyltransferase (DLST) antibody (1:1000) to control for contamination (bacterial and cytosolic components from the macrophage) (middle panel) and equal protein loading (lower panel), respectively. (C) dsRed-BCG-infected macrophages were stained for Cpn60.2 (green fluorescence) and MitoTracker Deep Red (red fluorescence). BCG red fluorescence was pseudo-colored as a blue signal to distinguish it from the MitoTracker signal. In the merge image (4×magnification), cyan color shows Cpn60.2 colocalization with BCG and yellow signal indicate the secreted Cpn60.2 co-localization with mitochondria. Large dotted circle indicates the macrophage cell boundary. Values are mean±s.d. of secreted Cpn60.2 colocalization with mitochondria in the vicinity of phagosomes observed in 50-60 cells from three independent experiments. Data in B and C are representative of three independent experiments.

Given the massive export of Cpn60.2 beyond mycobacterial phagosome, we consulted the web servers SLPFA (http://sunflower.kuicr.kyoto-u.ac.jp/~tamura/slpfa.html) and ESLpred (http://www.imtech.res.in/raghava/eslpred/) to define its destinations. SLPFA and ESLpred are frequently consulted for automated prediction of protein subcellular localization (Tamura and Akutsu, 2007) and both predicted mitochondria as the primary organelle target for Cpn60.2. Therefore, we reanalyzed additional EM sections of Cpn60.2-stained samples and observed frequent gold particles in the mitochondria (Fig. 2A). Thereafter, we purified mitochondria from Mtb-infected macrophages for western blot analyses and, as expected, Cpn60.2 was detectable in the mitochondrial fraction, consistent with the EM data (Fig. 2B). The membrane was then reprobed with antibody against Dos R, a secreted mycobacterial protein and it failed to detect any bands in the purified mitochondrial fraction excluding the probability of contamination from bacteria and cytosolic components from the infected macrophage. In other experiments, macrophages were infected with DsRed-BCG for 48 h then stained for Cpn60.2 and loaded with MitoTracker Deep Red. Preparations were analyzed by confocal microscopy and the results showed a clear co-localization of secreted Cpn60.2 with mitochondria in the vicinity of about 77.3±21.3% BCG-containing phagosomes (Fig. 2C). It was observed that the cleaved form of Cpn60.2 was enriched in BCG-infected macrophage mitochondria compared to the cytosolic fraction (Figs 1B and 2B). Since the Hip 1 mediated cleavage reduces the ability of Cpn60.2 to induce proinflammatory cytokine responses and thus allowing the pathogen to escape immune detection and to accelerate disease progression (Naffin-Olivos et al., 2014), the intra-mitochondrial enrichment of cleaved Cpn60.2 could be a clever survival strategy by Mtb to modulate host cell activities in a regulated manner.

How mycobacterial proteins cross the host endosomal membrane system remains an important question yet to be addressed. However, since Cpn60.2 has been shown to induce pores across planar lipid bilayers (Alder et al., 1990), one would suggests that Cpn60.2 might cross phagosome membrane bilayers by a pore forming process, reminiscent of *Listeria monocytogenes* toxin listeriolysin (Schnupf and Portnoy, 2007). On the other hand, given the high degree of similarity between bacterial and mitochondrial proteins regarding their function and targeting signals (Lucattini et al., 2004), mycobacterial Cpn60.2 is probably predisposed for targeting host mitochondria by a mechanism similar to that of the mitochondrial homolog Hsp60 chaperone.

### Cpn60.2 inhibits macrophage apoptosis

**Fig. 3.**
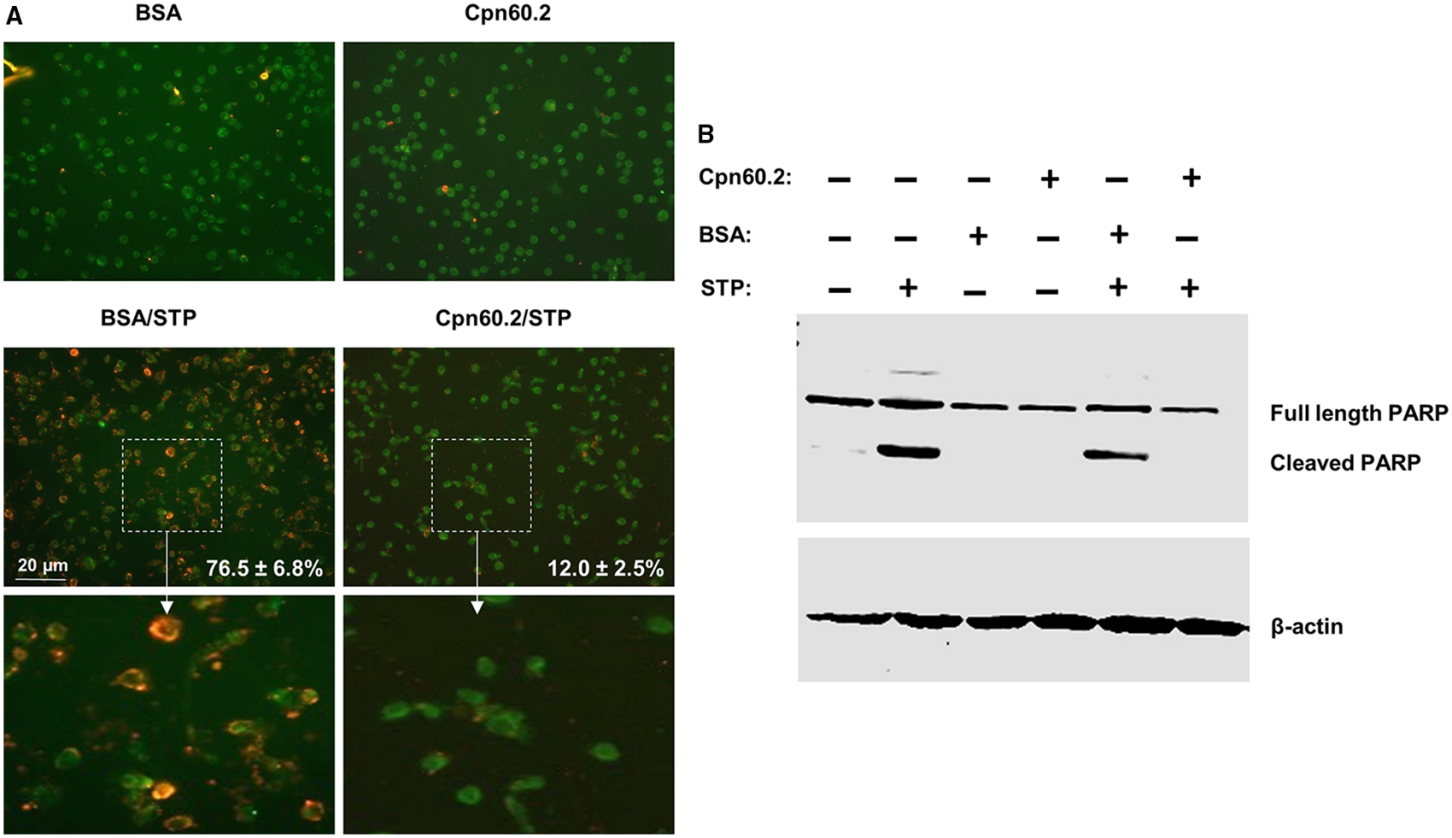
**Cpn60.2 inhibits macrophage apoptosis.** Macrophages were transfected with Cpn60.2 or FITC-BSA (control) then left untreated or stimulated with staurosporine (STP). (A) Cells were stained with Annexin V and confocal images are displayed as merges of green fluorescence (Cpn60.2 or BSA) and red fluorescence (Annexin V) channels. Lower panels show 4×magnification of random windows and numbers indicate mean values±s.d. for three independent experiments. (B) Total cell lysates were prepared and subjected to SDS-PAGE and western blotting with PARP antibody. Probing with β-actin antibody revealed equal protein loading in each lane. Data are representative of three independent experiments.

Mitochondria are pivotal in the regulation of the intrinsic program of apoptotic cell death (Green, 2005) and bacterial proteins targeting these organelles can either inhibit (Niu et al., 2010) or induce (Papatheodorou et al., 2006) apoptosis. Since Mtb evasion of innate immunity was shown to be associated with inhibition of macrophage apoptosis (Behar et al., 2010; Hmama et al., 2015), Cpn60.2 is probably blocking mitochondrion-dependent apoptosis. To examine this possibility, we first applied Annexin V cell surface staining for detection of phosphatidylserine (PS) translocation to the extracellular membrane leaflet, which is a marker of early stages of apoptosis (Vermes et al., 1995). Macrophages were transfected with recombinant Cpn60.2 or BSA (control) then treated with staurosporine to induce apoptosis. Treated cells were stained 24 h later for Annexin V and Cpn60.2 and examined by confocal microscopy. The images showed very low number of Annexin V-positive cells in Cpn60.2-transfected cells (12±2.5%) relative to the higher number of positive cells (76.5±6.8%) observed in control cells treated with FITC-BSA (Fig. 3A). These data indicate that Cpn60.2 blocks staurosporine-dependent translocation of PS to the cell surface. In a complementary series of experiments, we examined the extent of PARP cleavage, which is a typical apoptosis event that precedes nuclear fragmentation (Duriez and Shah, 1997). Cell lysates from transfected and treated macrophages as described above were subjected to western blotting with PARP antibody and results obtained (Fig. 3B) showed that relative to BSA transfected cells and cells treated with staurosporine alone, PARP cleavage is significantly reduced in cells transfected with Cpn60.2 and treated with staurosporine. These results along with the Annexin V data demonstrate that Mtb Cpn60.2 exerts an anti-apoptotic activity in the macrophage.

### Anti-apoptotic action of Cpn60.2 is dependent on its interaction with mitochondrial mortalin

The finding of Cpn60.2 translocation to the mitochondrion suggests that it might interact with and affect the function of apoptosis effectors. Thus we reasoned that if we can identify Cpn60.2 interacting protein within the Mtb cell, the human homologue of that protein could be the Cpn60.2 interacting protein within mitochondria. Thus, we consulted STRING (www.string-db.org), which is a powerful web resource of known and predicted protein-protein interactions, and requested Cpn60.2 interacting proteins inside the Mtb organism. STRING analysis predicted groS followed by dnaK to be the most probable functional partners (Fig. S1A and B). Thereafter we found that mitochondrial human HSPA9 (Hsp70, mortalin) has the highest similarity (62.6%) with dnaK (Fig. S2A and B). No human protein homologue was located in response to groS query.

**Fig. 4.**
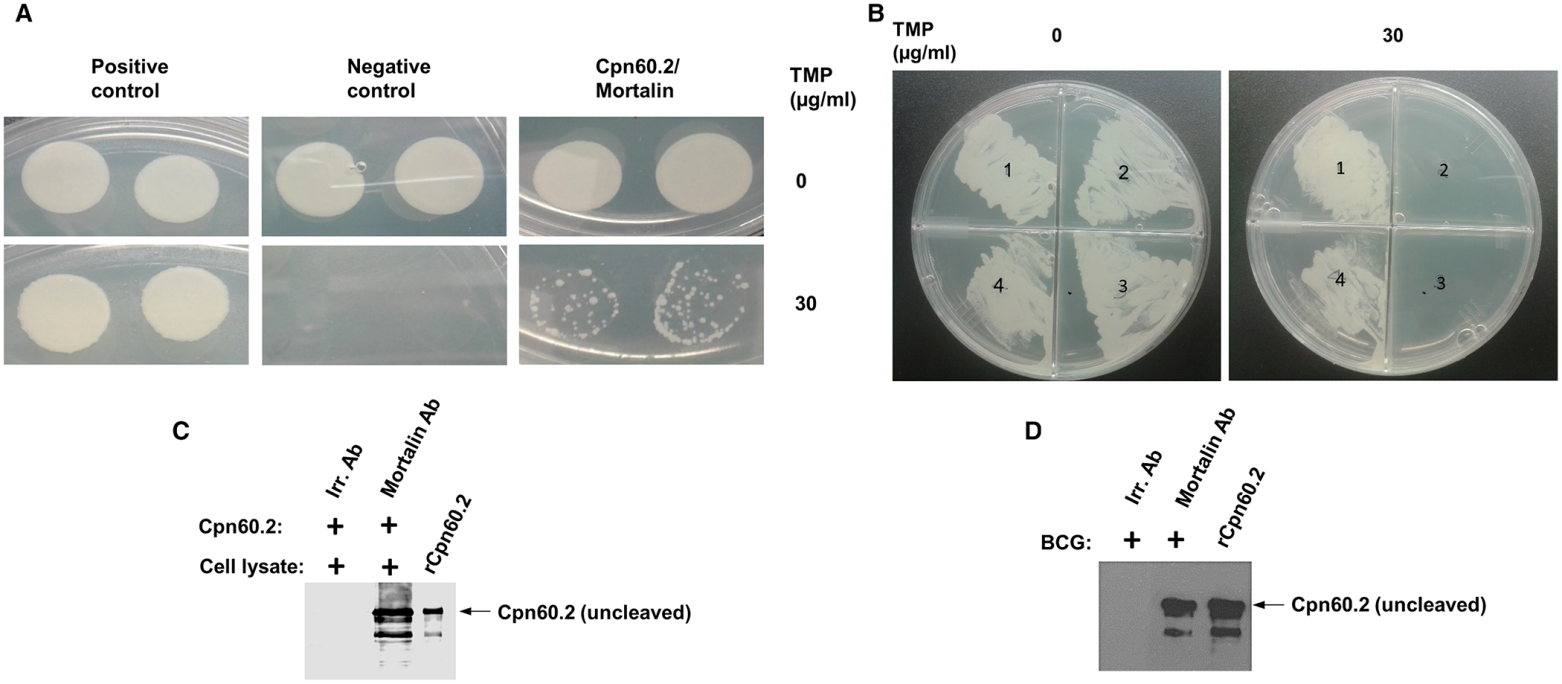
**Cpn60.2 interacts with mitochondrial mortalin.** (A) *M. smegmatis* expressing Cpn60.2-mDHFR F1,2 and mortalin-mDHFR F3 fusion proteins were plated on solid media containing trimethoprim (TMP). *M. smegmatis* expressing CFP-10 fused to F1,2 and ESAT-6 fused to F3 correspond to positive control. Negative control is *M. smegmatis* transformed with Cpn60.2-F1,2 and empty F3 constructs. The appearance of TMP-resistant bacterial colonies reflects the reconstitution of mDHFR as a result of protein-protein interaction. Experiments are shown in duplicates. (B) *M. smegmatis* co-transformed with Cpn60.2 (F1 and F2)/EFTM-F3 constructs and Cpn60.2 (F1 and F2)**/**Mortalin-F3 constructs were allowed to grow in the presence of trimethoprim as in A. *M. smegmatis* co-expressing Cpn60.2 (F1 and F2)**/**EFTM-F3 construct failed to grow in the presence of trimethoprim indicating the failure of interaction of Cpn60.2 with EFTM. (1) CFP 10_[F1,2]_**/**ESAT 6_[F3]_, (2) Cpn60.2_[F1,2]_**/**_[F3]_, (3) Cpn60.2_[F1,2]_**/**EFTM_[F3]_, and (4) Cpn60.2_[F1,2]_**/**Mortalin_[F3]_. (C) Equal amounts of macrophage lysates were incubated with rCpn60.2 protein and subjected to pull down assays with protein A/G magnetic beads conjugated with mortalin or irrelevant antibodies. Pulled down material was then subjected to western blotting with Cpn60.2 antibody as in Fig. 1B. (D) Protein lysates from BCG-infected macrophages were subjected to immunoprecipitation with mortalin or irrelevant antibodies then analyzed along with rCpn60.2 by western blotting with Cpn60.2 antibody as in Fig. 1B. Data are representative of three independent experiments.

To provide experimental proof of a direct interaction between Cpn60.2 and mortalin, we performed murine dihydrofolate reductase (mDHFR) protein fragment complementation assay in the intracellular milieu of *M. smegmatis* as described in the Materials and Methods section. As shown in Fig. 4A, co-expression of Cpn60.2 and mortalin bearing mDHFR fragments in *M. smegmatis* allowed for bacterial growth in the presence of trimethoprim antibiotic (mDHFR substrate) (TMP) as result of the reconstitution of functional mDHFR, dependent on physical association between Cpn60.2 and mortalin. Cpn60.2-mortalin interaction was slightly weaker than that observed between mycobacterial CFP-10 and ESAT-6 proteins (positive control), which naturally form a high affinity complex in mycobacteria (Renshaw et al., 2002). In order to show the specificity of Cpn60.2 interaction with mortalin, we have performed mDHFR assay of Cpn60.2 with a different mitochondrial protein, elongation factor Tu, mitochondrial (EFTM). Results showed that *M. smegmatis* co-transformed with Cpn60.2 (F1 and F2) and EFTM-F3 constructs failed to grow in the presence of trimethoprim, indicating the lack of Cpn60.2-EFTM interaction (Fig. 4B). In other experiments, we incubated recombinant Cpn60.2 with soluble fraction of macrophage lysates and were able to pull down mortalin associated with Cpn60.2 (Fig. 4C). Similarly, mortalin antibody was able to pull-down Cpn60.2 from lysates of BCG-infected macrophages (Fig. 4D). Taken together, these findings indicate a true physical association between Cpn60.2 and mortalin in infected macrophages.

**Fig. 5.**
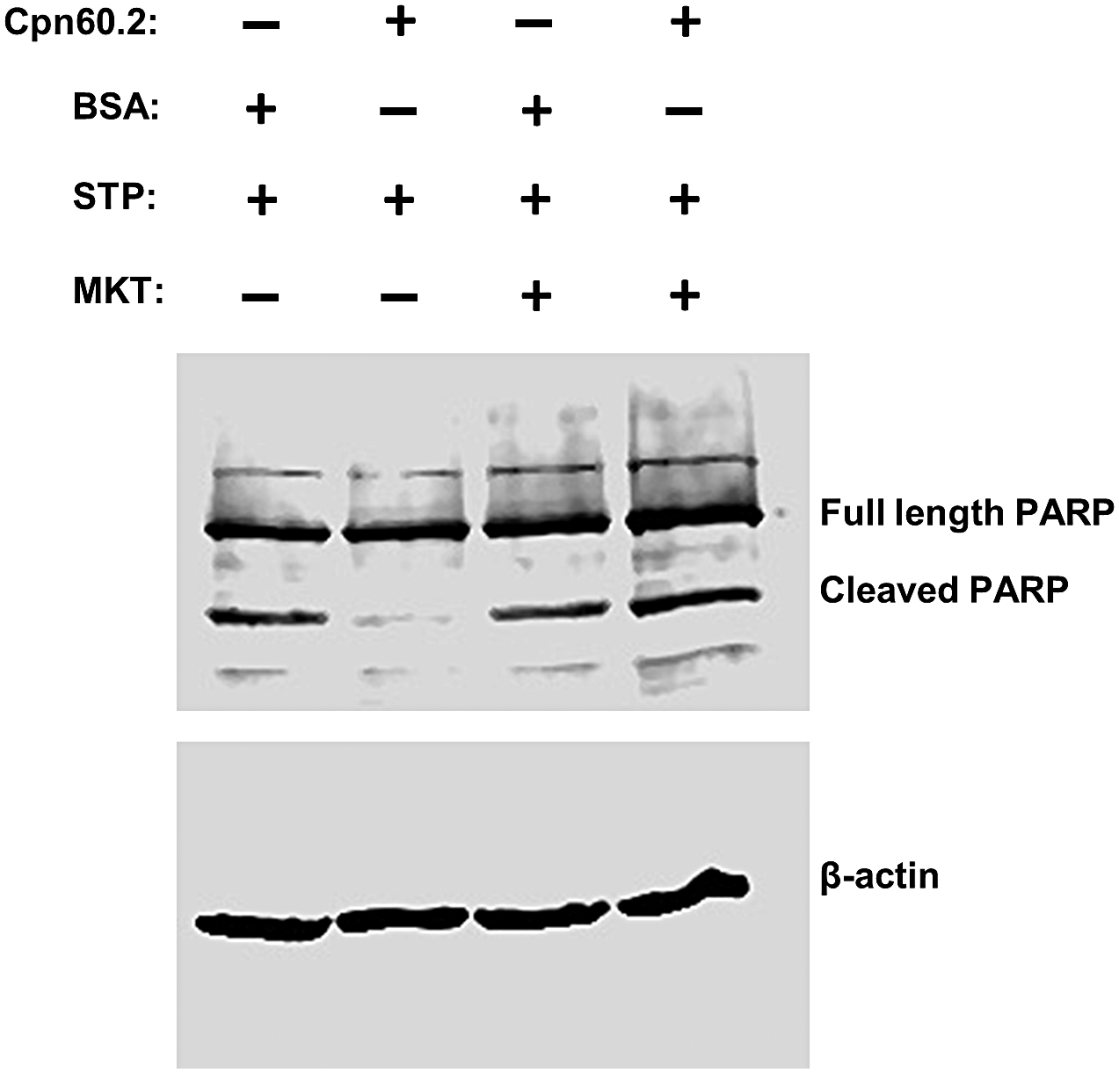
**Cpn60.2-mortalin interaction contributes to the anti-apoptotic potential of Cpn60.2.** Macrophages were transfected with Cpn60.2 or BSA (control) then left untreated or stimulated with staurosporine (STP). Where indicated, cells were pre-treated with 100 nM MKT-077 30 min prior to protein transfection. Cells were then subjected to PARP degradation assay as in Fig. 3B. Data are representative of three independent experiments.

Mortalin is primarily a mitochondrial protein (Yang et al., 2011). It protects cells from apoptosis and is overexpressed in cancer cells (Rozenberg et al., 2013). Thus, we suggested that, by virtue of its chaperone activity, Cpn60.2 binds to mortalin and increases its stability, and by doing so enhances its anti-apoptotic activity. The best approach to verify this hypothesis is to evaluate Cpn60.2 activity in cells lacking mortalin. Unfortunately, several attempts to knock out or knock down mortalin in the macrophage were unsuccessful, leaving us with the option of pharmacological inhibition. Thus, macrophages were treated with mortalin-specific inhibitor MKT-077 (Wadhwa et al., 2000) prior to Cpn60.2 transfection, and then subjected to staurosporine stimulation and PARP cleavage assays. Results obtained (Fig. 5) showed that MKT-077 treatment sensitizes Cpn60.2 transfected cells to staurosporine-induced PARP cleavage, indicating that Cpn60.2 interaction with mortalin rather blocks macrophage apoptosis.

### Conclusion

Many pathogens have evolved various effectors to interfere with apoptosis in order to persist intracellularly. The mechanistic study developed here demonstrates that Mtb uses Cpn60.2 as a potential virulence factor to disturb mitochondrion-regulated apoptosis via a direct interaction with mortalin. Given that Cpn60.2 is essential for Mtb growth, selective targeting of Cpn60.2 to inhibit its chaperone function in Mtb as well as its interaction with mortalin would open up exciting new avenues for TB drug development.

## MATERIALS AND METHODS

### Reagents and antibodies

AF568-Annexin V, MitoTracker Deep Red, Protein A/G magnetic beads and AF680 conjugated goat anti-rabbit IgG (A21109) were purchased from Invitrogen (Burlington, ON, Canada). FluidMAG-DXS beads were from Chemicell (Berlin, Germany). Mortalin (sc-133137) and β-actin (sc-1616-R) antibodies were from Santa Cruz Biotechnology (Santa Cruz, CA, USA). PARP antibody (9542) was from Cell Signaling (Denvers, MA, USA). FITC-conjugated goat anti-rabbit IgG (L43001) was from Caltag (Burlingame, CA, USA). DLST antibody (HPA003010), MKT-077 and paraformaldehyde (PFA) were from Sigma-Aldrich (St. Louis, MO, USA). Vir S antibody (ab22743) was purchased from Abcam (ON, Canada). Ultra-small goat anti-rabbit IgG (25101) was from Electron Microscopy Sciences (Hatfield, PA, USA). Staurosporine was purchased from Calbiochem (San Diego, CA, USA). Recombinant Cpn60.2 protein and Cpn60.2 antibody (kindly provided by Dr Richard W. Stokes, University of British Columbia, Canada) were described earlier (Hickey et al., 2010) and Dos R antibody was kindly provided by Dr Yossef Av-Gay, University of British Columbia, Canada.

### Mycobacterial strains, media and growth conditions

Mtb and *M. bovis* BCG were cultured and maintained as described previously (Sun et al., 2013). Briefly, Mtb strain H37Rv and *M.bovis* BCG (Pasteur 1173P2) were grown in Middlebrook 7H9 broth (BD Diagnostic Systems, Mississauga, ON, Canada) supplemented with 10% (v/v) OADC (oleic acid, albumin and dextrose solution; BD Diagnostic Systems) and 0.05% (v/v) Tween 80 (Sigma-Aldrich, St. Louis, MO, USA) at 37°C on a shaker platform at 50 rpm. Fluorescent Mtb and BCG expressing DsRed protein were described in a previous work (Sun et al., 2007). All transformations in *Escherichia coli* were done with strain JM109 and *E.coli* was grown in Luria-Bertani broth at 37°C in shaking cultures.

### Cell culture and infection

Pathogen-free THP-1 and RAW 264.7 cell lines were from ATCC (Manassas, VA, USA). RAW 264.7 macrophages were cultured in DMEM supplemented with 10% FBS and 1% each of L-glutamine, penicillin-streptomycin mixture, HEPES, non-essential amino acids (100× solution, StemCell). THP-1 cells were grown in RPMI 1640 supplemented with 1% each of L-glutamine, non-essential amino acids, penicillin-streptomycin mixture, HEPES and 10% fetal bovine serum. THP-1 cells were differentiated in the presence of phorbol myristate acetate (PMA; 25 ng/ml) at 37°C in a humidified atmosphere of 5% CO2 for 24 h. Macrophage monolayer was then infected at a multiplicity of infection (MOI) of 20:1 (bacilli to macrophages) according to the previously published protocol (Sun et al., 2013).

### Flourescence microscopy

Macrophages were infected with bacteria and at different time points post-infection, cells were fixed, stained and the coverslips were mounted on microscopic slides and examined by digital confocal microscope as described previously (Sun et al., 2010).

### Immunoelectron microscopy

Immunogold staining was performed as described earlier (Sun et al., 2013) at the EM Facility of the James Hogg Research Centre (Saint Paul Hospital, Vancouver, BC, Canada). Briefly, *M. tuberculosis*-infected macrophages were fixed with 4% paraformaldehyde, dehydrated in graded series of ethanol and water, and infiltrated with LR White resin. After polymerization at 50°C, 60 nm sections were cut with a Leica EM UC6 microtome (Leica Microsystems, Switzerland) and collected on nickel grids. The samples were then stained with anti-Cpn60.2 antibody followed by labeling with colloidal gold conjugated anti-rabbit IgG. Sections were then post-fixed in 2% glutaraldehyde and subjected to silver enhancement with Silver R-Gent SE-EM (Aurion, Wageningen, Netherlands). Samples were then washed with distilled water, stained in 2% uranyl acetate, washed again, air dried and examined with a Tecnai 12 electron microscope (FEI Company, Hillsboro, OR, USA).

### Cpn60.2 transfection and apoptosis assays

PMA-differentiated THP-1 cells (0.5×10^6^ cells per cover slip in 24-well plate) were subjected to magnetic transfection with Cpn60.2 or FITC-BSA (control) (200 ng) using PolyMAG beads according to the manufacturer's recommendations. At 4 h post-transfection, cells were treated with 50 nM staurosporine and stained 24 h later with Annexin V (1:20). Cells were then washed, fixed with 2.5% PFA and Cpn60.2-transfected cells were stained with Cpn60.2 antibody (1:100) followed by FITC-conjugated anti-rabbit IgG (1:3000). Cover slips were mounted on microscopy slides and analyzed by confocal microscopy. For PARP cleavage assay, 1×10^6^ cells, treated as above, were subjected to cell lysis with the Invitrogen extraction buffer and equal amounts of proteins (∼30 µg) were run on 9% SDS-PAGE gels. Protein bands were then transferred into western blot membranes and probed with PARP antibody (1:1000) followed by AF680-goat anti-rabbit IgG (1:10,000). Membranes were then imaged with Odyssey CLx^®^ imaging system (Li-Cor Biosciences, Lincoln, NE, USA).

### Immunoprecipitation of immunocomplexes

Recombinant Cpn60.2 protein was incubated with macrophage lysate for 2 h at 4°C and then added to protein A/G magnetic beads conjugated with anti-mortalin antibody (1 µg). The mixture was incubated for overnight at 4°C. The immunocomplexes were purified, resolved by 12% SDS-PAGE and immunoblotted on to a nitrocellulose membrane. The membrane was blocked with 5% milk powder, probed with an anti-Cpn60.2 antibody followed by incubation with secondary antibody, and imaged with the dual-color IR-excited fluorescence imager.

In order to pull-down Cpn60.2-mortalin complex from infected RAW cells, macrophage lysate prepared by sonication at 48 h post-infection was incubated with protein A/G magnetic beads conjugated with anti-mortalin antibody for overnight at 4°C. Then, the immunocomplexes were purified and immunoblotted with anti-Cpn60.2 antibody to look for a band corresponding to Cpn60.2.

### Mycobacterial protein fragment complementation (MPFC) Assay

MPFC assay was performed as described (Singh et al., 2006). In brief, Cpn60.2 gene was cloned into pUAB100 plasmid expressing murine dihydrofolate reductase (mDHFR) fragments F1 and F2 while mortalin was cloned pUAB200 plasmid expressing mDHFR fragment F3. *M. smegmatis* mc^2^ 155 was then co-transformed with both plasmids, and the co-transformants were tested for growth over a 5 days period on 7H11 kanamycin/hygromycin plates supplemented with 0 and 30 µg/ml of mDHFR substrate, trimethoprim. If protein-protein interaction occurs, it restores the assembly of mDHFR and its activity and therefore the growth of *M. smegmatis*.
